# Adhesive Precoated Bracket: Is It Worth Using? Long-term Shear Bond Strength: An *In Vitro* Study

**DOI:** 10.1055/s-0041-1740224

**Published:** 2022-02-17

**Authors:** Weerada Vorachart, Nonglak Sombuntham, Kulthida Parakonthun

**Affiliations:** 1 Orthodontic Division, Department of Pedodontics and Preventive Dentistry, Faculty of Dentistry, Srinakharinwirot University, Bangkok, Thailand

**Keywords:** orthodontic adhesive, adhesive precoated bracket, APC flash-free, shear bond strength, thermal cycling

## Abstract

**Objectives** The objectives of this study were to compare the long-term shear bond strength of conventional adhesive on metal brackets with that of adhesive precoated brackets *in vitro* and to evaluate the amount of adhesive remnant on the tooth surface after debonding.

**Materials and Methods** A total of 90 maxillary permanent premolars were randomly divided into two groups. The samples in the first group were bonded with metal brackets using Transbond PLUS Color Change Adhesive (TP), and the samples in the second group were bonded with Flash-Free adhesive precoated brackets (APC FF). The bonding techniques were performed, according to the manufacturer's instructions. The samples from each group were randomly divided into three subgroups with different thermal cycles ( *n* = 15). The shear bond strength (SBS) and adhesive remnant index (ARI) were measured and calculated.

**Statistical Analysis** Two-way ANOVA and Chi-square test were used to analyze the differences in the SBS and ARI between the groups, respectively.

**Results** The means of the SBS of the APC FF subgroups were significantly lower than those of the TP subgroups, except in the 10,000 thermocycle subgroups. Chi-square test showed no statistically significant differences between the groups and subgroups. An ARI score of 1 was the predominant score in both groups.

**Conclusions** This study found that the SBS of APC FF gradually increased with time and thermal aging did not affect the failure pattern.

## Introduction

Certain durations of orthodontic treatment are difficult to predict due to various factors, such as the severity of the initial malocclusion, the technique employed, the operator skill, and the need for patient cooperation. 1 Bracket failure is the second most common cause of longer treatment durations. 2 Many factors cause orthodontic bracket failure, including improper bonding procedures, adhesive failure, curing lamps, improper orthodontic appliances, masticatory loading, and some types of diets. 3

The bracket that is currently in use was developed from the edgewise appliance introduced by Dr. Edward H. Angle in 1925. To date, its design and characteristics have been improved by many companies. 4 The conventional bracket is bonded to the tooth surface with an adhesive resin. Total etching or self-etching techniques can be used to bond the brackets. 5 6 7 In 1996, the first light-cured precoated adhesive metal-based bracket was invented to eliminate the need for placing the composite on the bracket base. 8 The company claimed that this could reduce chair-time during the bonding procedure because there is no need to load the adhesive on the bracket. 9 10 11

Recently, a light-cured precoated adhesive, APC Flash-Free Adhesive (APC Flash-Free Adhesive Coated Appliance System, 3M Unitek) (APC FF), was introduced, claiming it could reduce the adhesive flash after bracket placement. The material consists of a compressible nonwoven matrix soaked in low-viscosity adhesive resin. Due to the lower viscosity and transparent color of the resin, when the bracket is pressed onto the tooth, the resin forms smooth edges seated on the tooth surface. Thus, it is not necessary to clean up any excess resin. The manufacturer applied APC FF to the Victory Series Low Profile bracket, which has a low profile, to reduce mucosal irritation and achieve better rotational control.

Ahmed et al reported the systematic review about *in vivo* bond strength studies of the orthodontic bracket-adhesive system. They found that there were six studies which passed the inclusion criteria and there was only one study that used APC metal brackets. 12 Studies on the shear bond strength (SBS) of APC FFs are still limited. Previous studies tested the SBS after bonding for 24 hours, which did not represent real orthodontic situations that take time to complete. 11 13 14 In 2019, González-Serrano et al studied the effect of thermocycling on SBS between APC FF and ceramic brackets. 15 However, few studies have compared the SBS between APC FFs and metal brackets with conventional loaded adhesives, which are widely used by many orthodontists. The purpose of this study was to compare the bond strength of APC FF and Transbond PLUS Color Change Adhesive (3M Unitek) (TP) with metal brackets under different thermal aging conditions. Long thermal aging conditions was the important point in this study to imitate the orthodontic treatment that takes at least a year. This present study also evaluated the amount of adhesive remnant on the tooth surface after debonding, which can explain the bonding failure in APC FF compared with the conventional bracket.

## Materials and Methods

### Samples

The sample size was calculated from program G*Power (Version 3.1.9.2). The effect size convention (f) 0.45 was used to calculate the sample size (total sample size = 80 or *n* = 13.33 per group).

After the sample size was calculated, the 90 extracted maxillary permanent premolars were collected from the dental clinics. In our study, maxillary permanent premolars were used because premolars are the most commonly extracted teeth during orthodontic treatment, 16 and the crown morphology of the maxillary first and second premolars appears to be more similar than that of the mandibular premolars. 17 All samples had to meet the inclusion criteria: (1) a sound tooth; (2) no carious lesion or crack line; (3) no adhesive or restoration or prosthesis on the buccal enamel; and (4) no previous exposure to chemical agents, such as hydrogen peroxide. The exclusion criteria were samples with (1) previous orthodontic treatment with fixed appliances and (2) developmental defects of the enamel. All of the extracted teeth were cleaned and stored in distilled water (ISO 3696:1987, grade 3) in a refrigerator for no more than 6 months, and the storage medium was replaced every 2 months, according to the International Organization for Standardization (ISO) norm specifications (ISO/TS 11405: 2015). 18

### Brackets and Adhesive

**Fig. 1:**
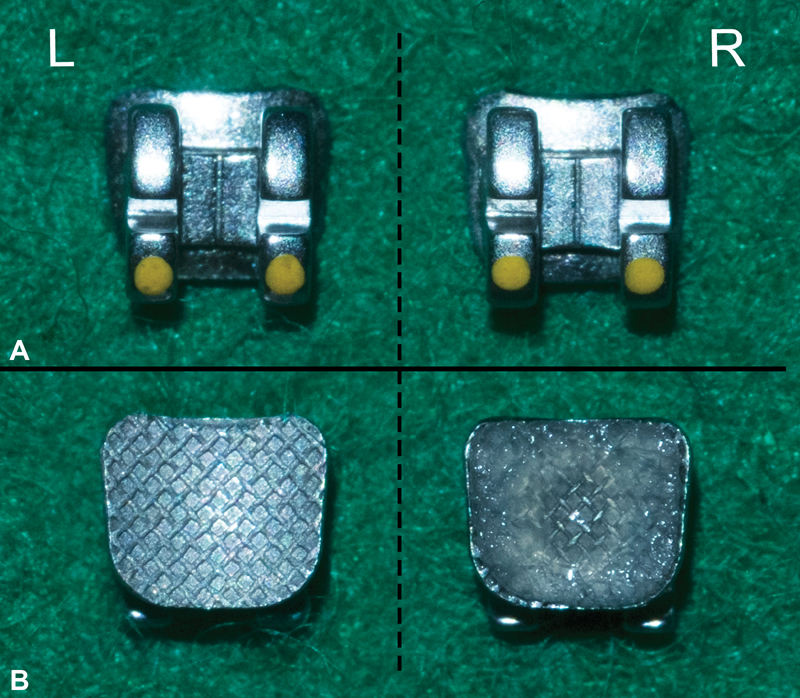
Physical features of the Victory Series Low Profile Bracket and the Flash-Free adhesive precoated bracket (APC FF): ( **A** ) shows a frontal view of both brackets, Victory Series (L) and APC FF (R); ( **B** ) shows the base of both brackets, Victory Series (L) and APC FF (R).

All teeth were randomly divided into two groups: (1) metal brackets (Victory Series Low Profile Brackets, 3M Unitek, United States) using Transbond PLUS Color Change Adhesive (TP, 3M Unitek, United States) ( *n* = 45) and (2) adhesive precoated brackets (APC FF, 3M Unitek, United States) ( *n* = 45). All brackets were MBT prescriptions, slot size 0.018 inches, and they had the same bracket base area of 9.9 mm ^2^ ( Fig. 1 ).

### Bracket Placement Procedure

**Fig. 2:**
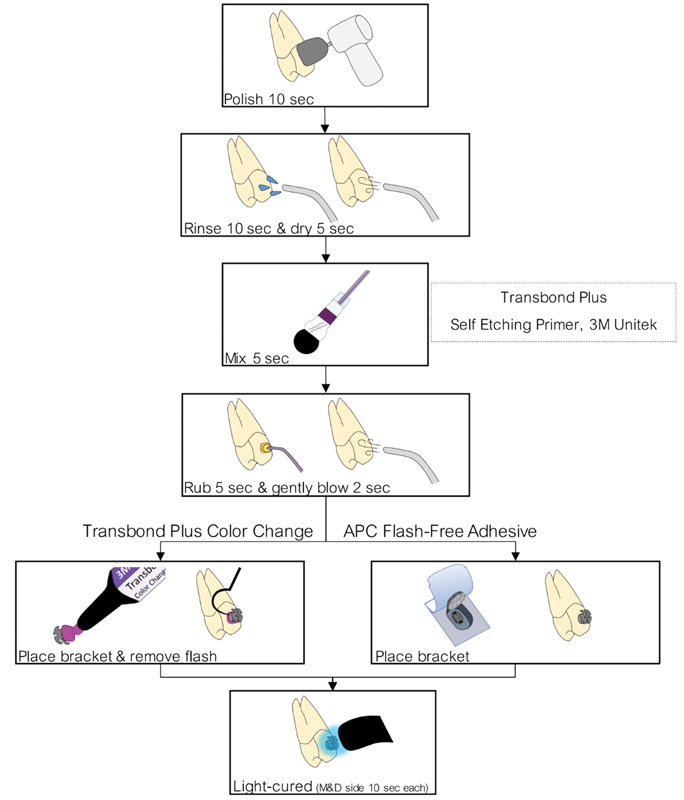
The bracket placement procedure in this study followed the manufacturer's recommendations.

All procedures were performed by the same operator (W.V.) to standardize the bonding procedure. The teeth were polished with fluoride-free pumice for 10 seconds, rinsed with water for 10 seconds, and air-dried for 5 seconds. The teeth were rubbed with Transbond Plus Self Etching Primer (3M Unitek, United States) for 5 seconds and gently air-dried for 2 seconds. For the first group, TP was pasted on a metal bracket base before being placed on the tooth surface at the FA point, which was the midpoint of the facial axis of the clinical crown. The bracket was pressed firmly on the tooth surface, and the excessive flash was removed with the explorer before light curing (2,000 mW/cm ^2^ , Mini LED SuperCharged, Acteon, France). The light tip was positioned on the mesial and distal sides of the bracket base to cure for 10 seconds on each side. For the APC FF group, after taking the brackets out of their packages, they were placed on the tooth surface in the same manner as the TP group and light cured immediately without removing any flash ( Fig. 2 ). All samples were stored in distilled water at 25 °C for 24 hours to complete the polymerization.

### Intervention

Samples from both groups were randomly divided into three subgroups ( *n* = 15): (1) stored in distilled water for 24 hours at 25 °C (the control), (2) thermocycling for 5,000 cycles or (3) thermocycling for 10,000 cycles by a thermocycling machine (TC301, Medical and Environmental Equipment Research Laboratory, KMITL).

According to Gale and Darvell, 10,000 thermocycles is approximately equivalent to 1 year of aging of a restoration in an oral cavity. 19 In this study, 5,000 and 10,000 thermal cycles in water (temperature between 5 °C and 55 °C, 20 seconds of dwell time, with respect to the ISO norm specifications ISO/TR 11405:1994 20 ) simulated the aging of the adhesives for 6 months and 1 year in an oral cavity, respectively.

**Fig. 3:**
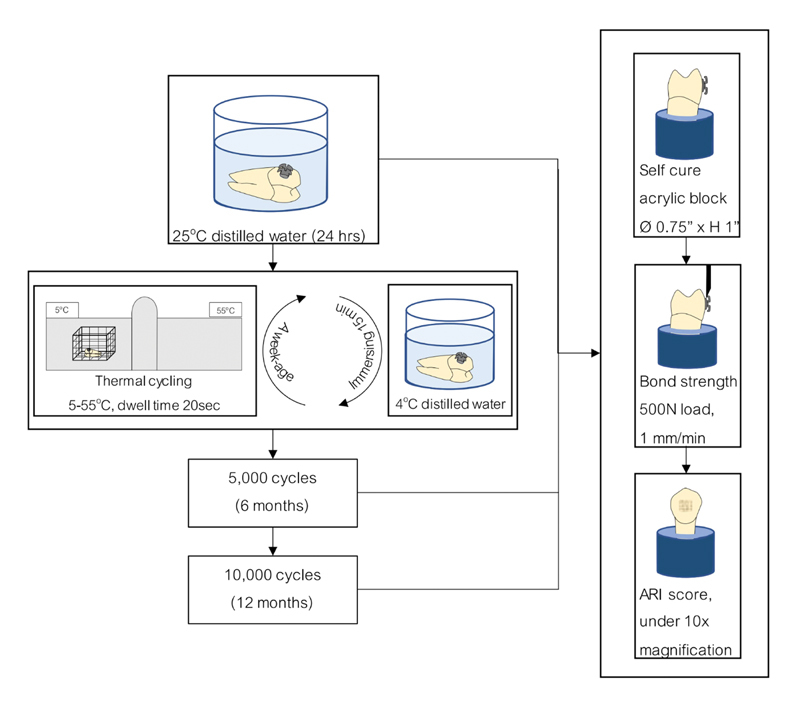
Intervention used in this study.

The specimens were immersed in 4 °C distilled water for 15 minutes every week during the thermal cycling, which was approximately every 200 cycles. After completing the 6-month and 1-year thermal aging, 15 specimens from each group were mounted in dental stone blocks (0.75 inches inside diameter and 1 inch long) and then placed in a universal testing machine (EZ-LX, Shimadzu). The failure load was measured with a 500 N load cell and a crosshead speed of 1 mm per minute until the bracket was detached from the tooth, following the ISO norm specifications (ISO/TS11405: 2015 18 ) ( Fig. 3 ). The loaded values were converted into the SBS in megapascals (MPa) by dividing the force values in Newtons (N) by the surface area of the bracket (9.9 mm ^2^ ). The bracket base was microphotographed, and its surface area was measured with ImageJ software.

The debonded specimens were evaluated for the amount of adhesive remnant on the tooth surface under 10X magnification by a stereomicroscope (SZ61, Olympus), and the ARI score by Artun and Bergland 21 was determined as follows:

0 = no adhesive left on the tooth.
1 = less than half of the adhesive left on the tooth.
2 = more than half of the adhesive left on the tooth.
3 = all adhesives left on the tooth.

### Statistical Analysis

A normal distribution and equality of variances of the SBS data were tested with the Shapiro–Wilk test and Levene's test, respectively. Two-way ANOVA was used to determine the influence of adhesive type and thermal aging on the SBS, and Tukey's test was used for posthoc comparisons. The ARI score was analyzed by Chi-square test. The SPSS 20.0 software package (SPSS, Chicago, IL, United States) was used for statistical analysis, and significance was defined as *p* < 0.05.

## Results

The results of this study showed that the type of adhesive had an effect on the SBS values. The mean SBS of APC FF tended to be significantly lower than that of TP, except in the 1-year subgroup.

The mean SBS of the APC FF group significantly increased with longer thermocycling durations when 24 hours and 1 year were compared. However, the mean SBS values of the TP group were almost the same in all subgroups. The mean SBS values of all groups are summarized in Table 1 .

**Table 1:** Mean SBS values ± standard deviation in MPa for the TP and APC FF groups after different thermal aging procedures ( *n* = 15)

Timing	Adhesive	
	TP	APC FF
24 hours	20.66 ± 4.88 ^a^	13.86 ± 4.14 ^b^
6 months	20.26 ± 4.10 ^a^	16.48 ± 2.18 ^bc^
1 year	20.53 ± 4.80 ^ad^	18.45 ± 2.95 ^cd^

Abbreviations: APC FF, flash-free adhesive precoated brackets; SBS, shear bond strength; TP, Transbond Plus Color Change Adhesive.

Note: The mean values with the same letter are not significantly different ( *p* > 0.05).

**Fig. 4:**
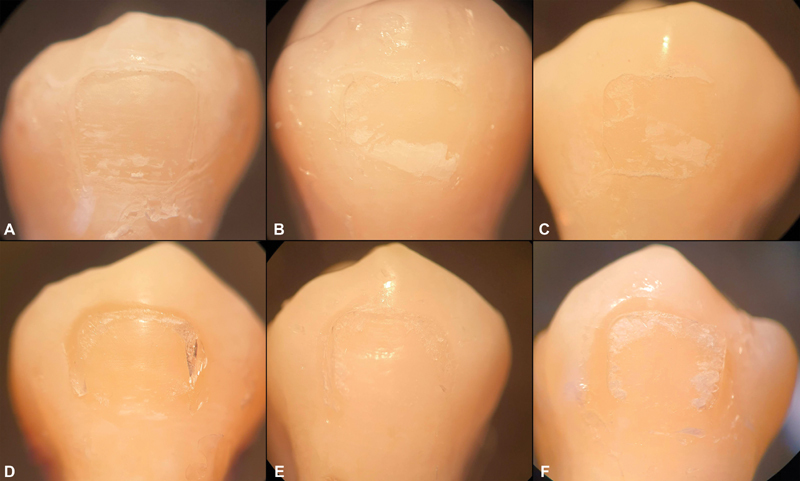
Surface of the specimens after debonding under a stereomicroscope (10X): from TP without aging ( **A** ), after 5,000 cycles ( **B** ) and after 10,000 cycles ( **C** ) of thermocycling; from APC FF without aging ( **D** ), 5,000 thermocycles ( **E** ) and 10,000 thermocycles ( **F** ). The ARI score from every group was 1.

Comparing the ARI score between the groups after debonding, Chi-square test showed no statistically significant differences among the adhesive groups or the thermocycling subgroups ( *p* > 0.05). Most of the specimens had less than half of the adhesive remaining on the tooth surface (ARI 1) in all groups ( Table 2 ). The ARI scores from each group are shown in Fig. 4 .

**Table 2:** Distribution frequency and percentages of ARI scores

Adhesive	Timing	ARI score (prevalence in %)			
		0	1	2	3
TP	24 hours	1 (6.7%)	11 (73.3%)	3 (20.0%)	0
	6 months	0	7 (46.7%)	8 (53.3%)	0
	1 year	0	12 (80.0%)	3 (20.0%)	0
APC FF	24 hours	0	11 (73.3%)	3 (20.0%)	1 (6.7%)
	6 months	0	10 (66.7%)	3 (20.0%)	2 (13.3%)
	1 year	0	11 (73.3%)	1 (6.7%)	3 (20.0%)

Abbreviations: APC FF, flash-free adhesive precoated brackets; ARI, adhesive remnant index; TP, Transbond Plus Color Change Adhesive.

## Discussion

The present study found that the SBS values of APC FF were significantly lower than those of TP except for the 10,000 thermocycling subgroup, which represented 1 year of aging in an oral cavity. The SBS of the TP group remained stable from the beginning until the end of the thermocycling process in this study, whereas the SBS of the APC FF group increased steadily with time. These findings were in contrast to a previous study, which found no significant differences between the TP and APC FF groups. 15 A possible explanation might be the difference in the material of the APC FF bracket. This study used the metal type, while the previous study used the ceramic type, which might be completely set immediately after light curing. The APC FF metal type might need more time to completely set than the APC FF ceramic bracket. Our study found that after 1 year, the SBS of APC FF was approximately 18.45 ± 2.95 MPa, which was similar to the TP group (20.53 ± 4.80 MPa). This result was comparable to a previous study that reported that the SBS of the APC FF and TP groups after 10,000 cycles of aging procedures was 18.1 ± 5.9 MPa and 20.1 ± 7.6 MPa, respectively. 15

As mentioned above, the bracket can be bonded to the tooth surface by using a total-etching or self-etching bonding system. Many previous studies concluded that the bond strength of the total-etching system was greater than that of the self-etching system. 5 6 7 In 1975, Reynolds proposed that SBS values greater than 6 to 8 MPa were sufficient for orthodontic purposes. 22 In this study, we used a self-etching primer, as in the study of Lee and Kanavakis, 11 and our results for the APC FF group were not much different from theirs (13.70 MPa). When compared with total-etching system studies, our TP SBS was slightly lower than the 23.7 MPa found in the study of González et al, 15 and our APC FF SBS was markedly lower than those obtained by Ansari et al, 13 Marc et al, 14 and González et al 15 (20.13 MPa, 21.77 MPa, and 24.00 MPa, respectively). This result confirmed that the self-etching bonding system provides a lower SBS than the total-etching system. However, the mean SBS values in this study were acceptable for orthodontic clinical usage. 22

Thermal cycling is widely used as an aging procedure for teeth or restorations by simulating intraoral temperature changes caused by drinking, eating, and breathing. The temperature fluctuation stresses the bond between the tooth and the resin, which may decrease the bond strength. 23 24 In the current study, the thermal aging method only had an effect on the APC FF group; the mean SBS values of APC FF increased significantly from 24 hours to 1 year, while TP remained constant at all times. A previous study tested the SBS of APC FF after thermocycling for 500 cycles and obtained results that were similar to ours at 24 hours and 6 months. 25 These results could be assumed to mean that the APC FF metal bracket requires more time to complete polymerization, while TP can complete cured within 24 hours after bonding. Another recent study found that 10,000 cycles of thermocycling significantly reduced the SBS of TP and APC FF, but there was no difference in SBS between 10,000 and 20,000 cycles when using a total-etch method with a ceramic bracket. 15

After debonding, the most prevalent ARI score of both groups was ARI 1, even after passing through the thermocycling phase. Although our results for the TP group differed from those of González et al, 15 who reported that thermocycling increased the ARI score (ARI 2 and 3), our results for the ACP FF group were similar to the ARI score of APC FF obtained by Lee and Kanavakis 11 and González et al. 15 They found that most APC FF cases left less than half of the adhesive on the tooth surface (ARI 1). The mode of bond failure in that study generally occurred at the tooth–adhesive interface. Leaving less adhesive behind on the tooth surface is preferable due to easier and safer removal of the residual resin, but it may cause enamel damage during the debonding process 26 27

However, the study of Ansari et al 13 and Grünheid and Larson, 28 who bonded APC FF ceramic brackets with a total-etching system, found that most APC FF cases had bond failure between the adhesive and bracket base (ARI 3). This may be explained by the differences in the experimental materials and methods used in each study. Ansari et al 13 used a smaller sample size than in our study and in González et al. 15 The study of Grünheid and Larson 28 was a split-mouth clinical study, and they placed brackets on one side of the maxillary premolars, canines, and incisors. At the end of the orthodontic treatment, they used a bracket debonding instrument to debond the brackets, unlike the use of a universal testing machine in an *in vitro* study. Further study should be performed for a longer thermocycling process to reflect the real situation of orthodontic treatment, and the specific process of polymerization of APC FF should be investigated.

## Conclusions

The SBS of TP with conventional brackets and APC FF metal brackets was sufficient for orthodontic bonding purposes. However, orthodontists should keep in mind that the SBS of the APC FF metal bracket increases with time. At 24 hours after bonding, the mean SBS of APC FF was significantly lower than that of TP; then, it increased to the same level as TP after 1 year of thermal aging, while TP exhibited a stable mean SBS value at all times. ARI 1 was the predominant score observed in this study, which indicated that thermocycling did not affect the failure pattern.
